# The Prospect of Identifying Resistance Mechanisms for Castrate-Resistant Prostate Cancer Using Circulating Tumor Cells: Is Epithelial-to-Mesenchymal Transition a Key Player?

**DOI:** 10.1155/2020/7938280

**Published:** 2020-03-30

**Authors:** Tanzila Khan, Kieran F. Scott, Therese M. Becker, John Lock, Mohammed Nimir, Yafeng Ma, Paul de Souza

**Affiliations:** ^1^School of Medicine, Western Sydney University, Campbelltown, NSW 2560, Australia; ^2^Medical Oncology, Ingham Institute of Applied Medical Research, Liverpool, NSW 2170, Australia; ^3^Centre of Circulating Tumour Cells Diagnostics & Research, Ingham Institute of Applied Medical Research, Liverpool, NSW 2170, Australia; ^4^South West Sydney Clinical School, University of New South Wales, Liverpool Hospital, Liverpool, NSW 2170, Australia; ^5^School of Medical Sciences, University of New South Wales, Kensington, Australia; ^6^Medical Oncology, Liverpool Hospital, Liverpool, NSW 2170, Australia; ^7^School of Medicine, University of Wollongong, Wollongong, NSW 2522, Australia

## Abstract

Prostate cancer (PCa) is initially driven by excessive androgen receptor (AR) signaling with androgen deprivation therapy (ADT) being a major therapeutic approach to its treatment. However, the development of drug resistance is a significant limitation on the effectiveness of both first-line and more recently developed second-line ADTs. There is a need then to study AR signaling within the context of other oncogenic signaling pathways that likely mediate this resistance. This review focuses on interactions between AR signaling, the well-known phosphatidylinositol-3-kinase/AKT pathway, and an emerging mediator of these pathways, the Hippo/YAP1 axis in metastatic castrate-resistant PCa, and their involvement in the regulation of epithelial-mesenchymal transition (EMT), a feature of disease progression and ADT resistance. Analysis of these pathways in circulating tumor cells (CTCs) may provide an opportunity to evaluate their utility as biomarkers and address their importance in the development of resistance to current ADT with potential to guide future therapies.

## 1. Introduction

Prostate cancer (PCa) is highly prevalent in the Western world; it ranks sixth among cancers in regard to mortality among men [1]. There were 1,276,106 new cases of PCa and 358, 989 deaths due to PCa worldwide in 2018 [2]. Despite dramatic improvements in five-year survival, mortality from PCa is poised to remain a major health problem due to increasing longevity, particularly in western countries. The most significant factors associated with morbidity and mortality are the development of metastatic spread to other organs, particularly bone, and emerging resistance to therapy.

On the molecular level, PCa is almost always initially driven by excessive signaling through the androgen receptor (AR) pathway (reviewed in [3]). Consequently, men with metastatic PCa will be offered androgen deprivation therapy (ADT) as the primary treatment. After a median of around 18–24 months, the disease tends to become resistant to hormonal manipulation and progresses towards so-called metastatic castration-resistant prostate cancer (mCRPC). In mCRPC, the concentration of the current blood-based clinical PCa biomarker, prostate-specific antigen (PSA), continues to increase over time. As PSA is regulated *via* AR signaling, this suggests, in general, the common ongoing involvement of AR signaling in disease progression to mCRPC [4–7]. Abiraterone [8, 9] and enzalutamide [10, 11] have been developed to be used for mCRPC, as “second-generation” ADT treatments, and responses are generally good, but a median progression-free survival of 5.6 months [8] suggests resistance to treatment once again supervenes. Indeed, despite the difference in mechanisms of action, cross-resistance between enzalutamide and abiraterone is very common [8, 12–14], suggesting the development of true hormone resistance following second-line ADT therapy, as opposed to castrate resistance. Thus, androgen signaling through AR within the context of the oncogenic effect of other signaling pathways remains an important area of research as there are, yet, no effective treatments or markers for true hormone resistance. Here, we review the involvement of two critical signaling pathways, the phosphatidylinositol-3-kinase/AKT (PI3K/AKT) and Hippo/YAP pathway, which interact with the AR pathway in mCRPC and which have links to epithelial-to-mesenchymal transition (EMT). EMT is thought to play an important role in the development of both metastasis and therapy resistance [15, 16]. Our literature research indicates that the analysis of circulating tumor cells (CTCs) isolated from PCa patients may allow CTCs to be used as a tool to define how these signaling pathways interact with the AR pathway to cause ADT resistance and thereby investigate the mechanism by which these pathways may contribute to castrate resistance. In addition, CTCs may thus emerge as a useful PCa biomarker for personalized therapy.

## 2. Circulating Tumor Cells and EMT in Metastasis

**Figure 1:**
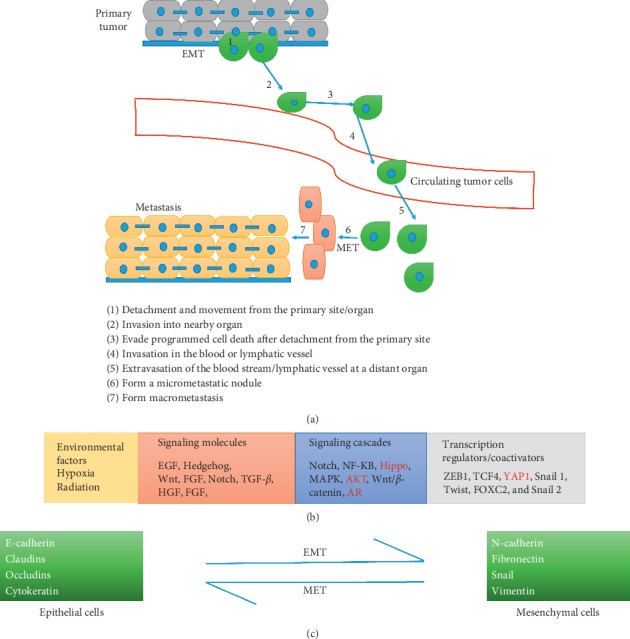
EMT in cancer metastasis. (a) Schematic representation of the role of EMT in cancer metastasis. (b) A cascade of transcriptional regulation underlies the transition from an epithelial to a mesenchymal phenotype, and (c) during EMT, epithelial markers are downregulated while mesenchymal markers are upregulated.

Metastasis in PCa is integrally linked to mCRPC. At the cellular level, metastasis involves a sequence of steps, and current evidence suggests that EMT and the reverse process mesenchymal-to-epithelial transition (MET) (reviewed in [17]) are important mechanisms by which tumor cells migrate and reestablish themselves at distant sites. Cancer cells are believed to lose their tight adhesion to neighboring cells and become more mobile when undergoing EMT, which, in turn, favors their ability to shed from the tumor mass, intravasate into the bloodstream, and thus become CTCs. MET, on the other hand, is thought to aid CTCs after leaving the vascular system to be able to settle in other tissues and form new tumors [18, 19] (Figure 1). Thus, CTC numbers have been recognized as a marker of metastatic disease, and importantly, EMT markers have been screened for in patient CTCs including those of 54 patients with PCa, 53% of these patients had advanced metastatic disease and intermittent epithelial-to-mesenchymal phenotype of CTCs correlated with metastasis in these patients, while another study found that the mesenchymal CTC phenotype correlated with increased rates of progression to CRPC in a cohort of 108 PCa patients recruited with high volume metastatic disease at hormone-sensitive disease stage and longitudinally followed during the study [20–22].

Metastatic spread of cancer is thought to involve different stages (Figure 1(a)) in which cancer cells (i) lose cell-cell tight junctions and detach from the primary site/organ, (ii) penetrate the basal lamina and enter nearby tissue, (iii) evade programmed cell death normally induced by loss of substrate adhesion (anoikis), (iv) breach blood or lymphatic vessels and migrate to other sites *via* blood/lymphatic circulation, (v) leave the bloodstream or lymphatic vessels at distant organs, (vi) form a micrometastatic core, and finally (vii) adjust and reprogram the surrounding stroma to form detectable macrometastases [23]. At a molecular level, EMT has been implicated in various cancers, including PCa. In the development of mCRPC, it has been proposed that activation of transcription factors (TFs) results in the loss of epithelial properties and acquisition of mesenchymal characteristics as well as the change of cell shape, leading to enhanced invasion and increased mortality [24, 25].

EMT is inducible by environmental factors such as radiation or hypoxia (Figure 1(b)), and there is accumulating evidence that radiation or chemotherapy, used to treat earlier stage PCa, may induce EMT changes [26, 27]. Hypoxia induces the production of hypoxia-inducible factor (HIF), and HIF-1*α* stimulates transcription factors (TFs), such as Snail and Twist, to trigger EMT [28, 29]. EMT then results from activation of a mesenchymal transcriptional program induced by specific transcription factors (EMT-TFs) [26]. Mechanistically, central EMT-TFs ZEB1, Snail, Slug, and Twist along with other TFs such as TCF4 and FOXC2 suppress the expression of key epithelial markers such as cytokeratin, E-cadherin, occludin, and claudin while causing upregulation of mesenchymal markers such as N-cadherin, fibronectin, and vimentin, which enable cancer cells to be more motile and consequently more aggressive (Figure 1(c)).

Regulation by signaling cascades and signaling molecules including EGF, Hedgehog, Wnt, FGF, Notch, TGF-*β*, and HGF in turn induces signaling *via* NF-*κ*B, MAPK, PI3K/AKT, or Wnt/*β*-catenin pathways to regulate EMT-TFs and ultimately induce EMT phenotypic changes. More recently, the Hippo pathway has been implicated in regulating EMT *via* its downstream transcriptional modulator Yes-associated protein (YAP) and the transcriptional coactivator TAZ [28, 30–38]. Importantly, there is evidence in the literature that these pathways can be successfully analysed in CTCs even though in some cases these analyses may not have yet been reported for PCa CTCs.  Table 1 summarises some of the evidence implicating signaling pathways in EMT of PCa as well as the analysis of these pathways in CTCs mainly from other cancers. CTC studies from other cancers are included because they indicate the feasibility of investigating these pathways in PCa CTC.

## 3. Clinical Relevance of EMT Markers in PCa

Several studies have assessed EMT markers for their clinical importance at various stages of human PCa.  Table 2 shows typical EMT markers detected in PCa tissue. A possible clinical utility of these EMT markers at different phases of the disease is suggested by their prognostic correlation with both recurrence-free and overall survival. For example, EMT markers Twist and vimentin as measured by immunohistochemistry in radical prostatectomy samples are independent markers for biochemical recurrence as defined by a resurgence in serum prostate-specific antigen (PSA) levels postsurgery [84, 90]. A recent study found that Cathepsin L (Cat L), which is an EMT-associated target of the EMT-TF Snail, may be a biomarker of PCa progression [83]. In addition, loss of membrane-bound E-cadherin staining appears to be linked with higher Gleason score, advanced clinical stage, and poor prognosis in PCa [91]. EMT markers such as Zeb1, E-cadherin, and vimentin play important roles at different stages of disease progression from primary tumor stage 2 to CRPC. In CRPC, increased expression of Zeb1 correlated with decreased survival [84]. Further, in a study of 108 patients with newly diagnosed castrate-sensitive PCa, expression of mesenchymal markers in CTCs at baseline was found to be an independent prognostic factor that was predictive of time to progression to CRPC following standard ADT. Patients who had mesenchymal CTCs at baseline showed a significantly shorter time to progression to CRPC than patients without CTCs or patients whose CTCs were negative for mesenchymal markers [21]. Several studies show that E-cadherin suppresses invasion and metastasis *in vitro*, and consistent with these findings, E-cadherin staining in tumor tissue correlates with longer overall survival [84]. However, the relationship of E-cadherin to metastasis is not clear in all cases since, in a recent study, it has been shown that loss of E-cadherin reduced metastatic potential in invasive ductal carcinomas [92], suggesting that E-cadherin plays opposing roles in tumor progression by suppressing cancer cell invasion while promoting metastasis. Nonetheless, on balance, the data suggest that EMT markers may have predictive value with respect to recurrence and overall survival both in tissues and in CTCs [84]. Different studies show that E-cadherin suppresses invasion and metastasis. However, in a recent study, it has been shown that loss of E-cadherin reduced metastatic potential in invasive ductal carcinomas [92].

## 4. AR, ADT, EMT, and Drug Resistance

**Figure 2:**
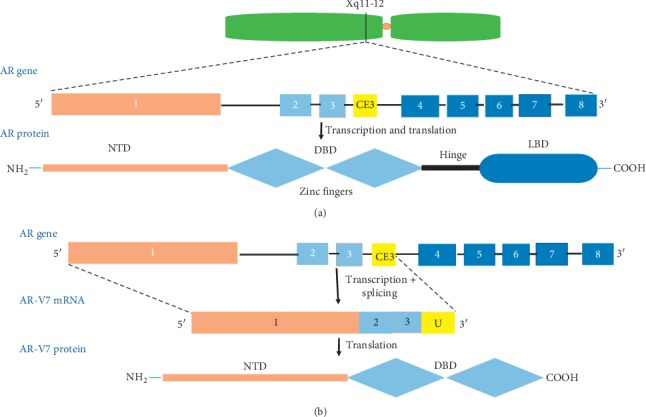
AR and AR-V7 gene and protein. The schematic indicates (a) the structural organisation of the AR gene and protein (NTD: amino terminal domain; DBD: DNA-binding domain; LBD: ligand-binding domain). (b) The transcription and translation of the AR-V7 protein including the exon/intron composition of the AR, highlighting the cryptic exon CE3 (middle) and domains of the AR retained in the AR-V7 protein (bottom).

The AR, located on the X chromosome (Figure 2(a)), is a hormone-dependent transcription factor [93]. In the unstimulated state, the receptor is cytoplasmic and bound by heat-shock proteins [94]. When its ligand, dihydrotestosterone (DHT) or testosterone, binds *via* the AR ligand-binding domain (LBD) (Figure 2(a)), a structural change results in the detachment of AR from the heat-shock protein 90 (HSP90) complex, homodimerization of the receptor, and nuclear translocation.

In the nucleus, AR acts as a transcription factor by binding to androgen-response elements (AREs) in the promoter region of androgen-regulated genes [95, 96]. AR transactivates genes which are responsible for cell growth, differentiation, and cell survival [97]. Consequently, increased AR signaling can potentially transform normal prostate cells into malignant PCa cells. Moreover, it has been shown that ADT therapy can select for cancer cells with further increased AR activity, for example, due to AR gene amplification [98].

The expression of alternative AR splice variants has been proposed as a mechanism underlying resistance to ADT [99, 100]. Most splice variants result in the translation of a truncated AR protein lacking a functional C-terminal LBD but containing a functional transactivating N-terminal domain. Without being capable of binding ligand, the resulting proteins are constitutively active as transcription factors and able to promote expression of certain target genes [97, 101]. At least 20 splice variants of AR have been identified in human prostate tissue and have been implicated in the development of mCRPC [101–104]. Amongst AR variants, AR-V7 is highly expressed in mCRPC and is the most frequently disease-associated variant identified in the clinic [105, 106]. The AR-V7 transcript results from alternative splicing of the AR gene such that the transcript contains exons 1, 2, and 3 together with a cryptic exon 3E (CE3) resulting in a truncated transcript (U), resulting in premature transcriptional termination (Figure 2(b)). AR-V7 is constitutively active irrespective of androgen binding, which is a proposed mechanism of escape from ADT [107, 108].

There is no clear consensus with respect to the role of androgen signaling in the regulation of EMT. An early study using cell lines showed that androgen stimulation promoted EMT in both LNCaP and PC-3 cells but that there was an inverse relationship between AR receptor levels and androgen-mediated EMT marker expression and EMT-associated cytoskeletal changes. Low levels of AR induced by shRNA promoted PCa cell metastatic ability by inducing EMT while high levels did not [109]. In contrast, a recent study has shown that AR mRNA and protein expression is higher in metastatic tumor tissues than in primary tumors and increases with tumor stage and Gleason score. Patients with higher AR expression showed shorter recurrence-free survival, indicating a positive association between AR expression and tumor progression. Further, knockdown of AR using siRNA in C4-2B cells suppressed functional markers of EMT, *viz* cell migration and invasion, and mesenchymal marker proteins associated with EMT, while increasing the epithelial marker E-cadherin. These effects were recapitulated by treatment with the antiandrogen bicalutamide [39]. Thus, it appears that AR stimulation induces or suppresses EMT in cell culture in a cell-type-dependent fashion.

Studies with both normal mouse prostate and human prostate tumor models in mice have shown that androgen deprivation through surgical castration, while suppressing tumor growth, induces mesenchymal markers of EMT and markers of a stem cell phenotype, while suppressing epithelial markers. These changes were also seen in tissues of patients treated with ADT [110], supporting the view that AR signaling suppresses EMT, while ADT promotes it.

In further support of this view, ADT with enzalutamide in C4-2 cells, but not in PC-3 cells, induced EMT markers in a Snail-dependent fashion. Induction of EMT required both suppression of AR signaling and activation of Snail. Interestingly, Snail was downregulated by androgen in AR expressing C4-2 and VCaP cells but again, not in PC-3 cells. Importantly, the inverse correlation between AR signaling and Snail expression observed in C4-2 xenografts and castration-resistant patient-derived metastases in mice and in clinical samples supports the view that the induction of EMT is an adaptive response to ADT with enzalutamide [40]. ADT may favor acquisition of stem cell and EMT characteristics, expression of oncogenes, or suppression of tumor suppressor genes in AR-positive PCa cells, implying that mCRPC at least in part is achieved through EMT [41, 110–114].

Other data suggest that AR splice variants are involved in the development of drug resistance in PCa [105, 115–117]. One corollary of this hypothesis is that inhibition of the AR variants or their specific function might lead to reversal of EMT phenotype and that might in turn inhibit tumor spread [41, 118]. Overall, however, this area remains understudied, and more data are needed to fully understand how the AR pathway and its manipulation during therapy may regulate EMT and thus potentially metastasis. Since mCRPC is ultimately the principal cause of death in many patients, the fundamental biological processes for the development and establishment of mCRPC need to be understood [119]. It is noteworthy that there is now mounting evidence in CTCs that the expression of EMT markers is associated with mCRPC [120, 121], highlighting the potential benefit in the analysis of CTCs to address the role of AR in metastasis and drug resistance.

## 5. Akt Pathway in mCRPC

As indicated above, due to the hormone-independent nature of mCRPC, it is unresponsive to all current forms of ADT. At this stage, AR expression may even be completely lost [122–124], raising the question as to how survival and proliferation of PCa cells occur at this stage. The main oncogenic signaling pathway implicated at this juncture is the PI3K/AKT-pathway, predominantly activated through frequent functional loss of the inhibitory tumor suppressor phosphatase and tensin homolog (PTEN), which is less common in localized PCa (20–30%) but becomes more dominant and is found in up to 50–60% of mCRPCs. The result is uncontrolled, oncogenic Akt signaling (reviewed in [125, 126]). The PI3K/AKT and AR pathways are highly networked with both positive and negative feedback loops [125], and in mCRPC, current literature indicates that negative feedback dominates. That is, inhibition of one pathway leads to reciprocal activation of the other [127–130]. Carver and colleagues have elucidated part of this interaction, demonstrating that the AR reduces AKT activation through the intermediary PHLPP, while AKT can transcriptionally downregulate AR output via HER kinase activity [127]. The exact role of PTEN in mediating this interaction is controversial. On the one hand, PTEN deletion has been associated with AKT activation and reduced AR levels [128, 131], and on the other hand, it may independently increase AR gene expression by removing transcriptional repression [130, 132–134]. Given the interconnected signaling network, outcomes of AR and AKT signaling or silencing may affect overall outcomes in a context-specific fashion, which is likely dependent on the presence and activity of other proteins that can affect the balance of feedback loops. For example, it has been shown that AR can transcriptionally repress PTEN expression in PCa cells while it increases PTEN expression in breast cancer cells and the report suggested this may be due to tissue-dependent availability of transcriptional cofactors [135]. Moreover, ADT may also affect the balance in these interconnected signaling pathways. Importantly, loss of *PTEN* has been associated with EMT driven through the AKT pathway or in cooperation with RAS signaling; thereby, lack of PTEN function could promote metastasis [136, 137].

## 6. Hippo Signaling Pathway and Its Role in CRPC and EMT

**Figure 3:**
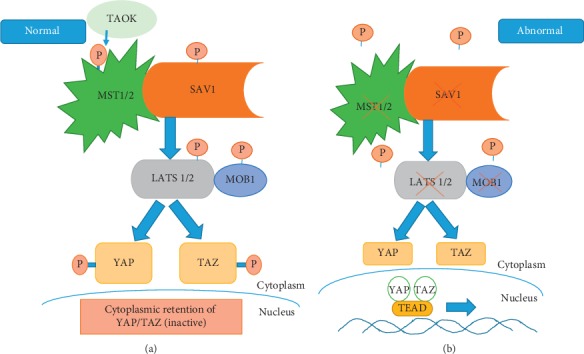
Hippo signaling pathway. Active Hippo signaling represses YAP and TAZ *via* phosphorylation (a), while inactive Hippo leads to dephosphorylation, nuclear translocation, and thus activation of TFs (b). The crossed out symbol indicates pathway members frequently lost in cancer.

As indicated above, several signaling pathways may contribute to the induction of EMT and ultimately metastasis, with the AKT pathway of importance in the context of PCa. More recently, the YAP1 transcriptional coactivator regulated by the Hippo pathway has emerged as an important player in this scenario and in regulating PCa cell motility [138]. In the context of gastric cancer, PTEN inactivation has been proposed to link the Hippo and PI3K/Akt pathways to promote cancer development and tumorigenesis [139]. In normal tissue, the Hippo signaling pathway appears central to cell growth control and limits organ size by coordinating cell proliferation, growth, and death [140]. Different signals like cell polarity, cell-cell contact, extracellular matrix characteristics, and stress can result in the activation of the Hippo pathway (reviewed in [141]). Hippo signaling through a kinase cascade results in phosphorylation of oncogenic cotranscription factors known as YAP and TAZ, promoting their cytoplasmic retention and proteasomal degradation [142–144] (Figure 3).

Inactivation of the Hippo pathway allows for YAP and TAZ activation *via* dephosphorylation, which is required for translocation into the nucleus. Although TAZ and YAP lack intrinsic DNA-binding domains, they are recruited by and enhance the activity of other transcription factors at their target promoters [145, 146].

Hippo signaling can act as a tumor suppressor. Functional impairment of Hippo signaling is often due to the loss of MST1/2 or LATS1/2 function or due to *YAP1* gene amplification. YAP1 is the most studied YAP isoform, and aberrant YAP1 activation is associated with the etiology of various malignancies including stomach [147], thyroid [148], lung [149], colon [150], head and neck [151] ovarian [152], liver [153], and prostate cancer [154].

Most interestingly, YAP1 and AR directly interact in PCa cells. One study demonstrates that unlike in hormone-sensitive prostate cancer cells, YAP1-AR interactions are androgen-insensitive and may cause resistance to enzalutamide in mCRPC cells. The WW/SH3 domain of YAP1 most likely facilitates the interaction with the AR amino terminal domain (NTD) [155].

One study proposes that increased nuclear YAP1, possibly due to the loss of Hippo signaling, may lead to increased complex formation between AR and YAP1 leading to androgen-independent binding of the complex to AREs in AR-driven promoters resulting in aberrant AR target gene expression possibly promoting mCRPC [58].

Importantly, YAP has been shown to promote metastasis through several mechanisms including EMT, and there is some evidence that the PTEN-AKT axis is involved in YAP1-induced EMT [145, 156, 157]. The underlying mechanisms of EMT regulation by YAP are still emerging, but given the role of YAP as a transcriptional coregulator, it is not surprising that the pathways centrally involve EMT-TFs. Critically, YAP1 has been shown to network with the main EMT-TFs. For instance, high glucose-induced polyubiquitination of PTEN results in alteration of its phosphatase targets, including an increased focus on dephosphorylation and activation of EMT regulators such as Twist, Snail, and YAP1 [158]. YAP1 was also reported to drive EMT and likely NSCLC metastasis by TEAD-dependent transcriptional induction of *SLUG* [159]. Focusing on YAP's role in osteoblast differentiation, one study identified two links between YAP and Snail/Slug. In Snail/Slug-null skeletal stem/stromal cells, the levels of both YAP and TAZ were reduced *via* protein degradation due to activation of the Hippo pathway, while direct interaction of YAP with Snail and with Slug was shown to alter YAP/TEAD transcriptional activity [160]. Another study found that Twist-induced EMT in breast cancer cells is dependent on TAZ activity. The mechanism involved increased expression of the Hippo pathway inhibitors PAR-1 and PAR-3, which drive TAZ nuclear localisation. One would expect that YAP nuclear localisation may also be induced *via* PAR-1/-3 in this context, although this was not examined [161]. Another study revealed that increased extracellular matrix stiffness can induce EMT in breast cancer cells and that blocking *β*1-integrin-mediated matrix stiffness prevented both Twist and YAP nuclear translocation albeit, interestingly, by different mechanisms [162].

In epithelial cells, cells are connected to each other by membrane structures called tight junctions, adherens junctions, and desmosomes. Any dysregulation in these junctions is implicated in metastasis and EMT [163, 164]. Zona occludens-1 (ZO-1) is a tight junction protein that is present in normal epithelial cells. Though not yet studied in PCa, in melanoma, lung cancer cells, and breast cancer, ZO-1 expression correlates with invasion properties of cancer cells [165–167]. One study found that YAP overexpression resulted in downregulation of ZO-1 and induced metastasis through EMT in NSCLC [159].

YAP (but not TAZ) has been shown to interact directly with ZEB1 and, remarkably, this interaction turns this transcriptional repressor into an activator. This is highlighted by the fact that ZEB1-mediated CDH1 (E-cadherin) repression is independent of YAP binding. Critically, gene upregulation by the ZEB1-YAP complex correlated with gene expression signatures of claudin-low breast cancer, a breast cancer subtype overall exhibiting an EMT phenotype. More importantly, ZEB1-YAP complex-mediated gene expression was related to poor patient survival in hormone-independent breast cancers and linked to drug resistance and metastasis [168]. ZEB1 is known to repress several EMT-related miRNAs including miR375, which is associated with an epithelial phenotype. Nevertheless, miR375, a known YAP target, is commonly overexpressed in PCa and in fact has been indicated as a plasma marker of PCa. The suggested mechanism by which miR375 supports an epithelial phenotype is via feedback regulation, such that it targets and suppresses YAP transcript and thus YAP protein levels and thereby reversing EMT in PCa cells. Surprisingly however, high plasma miR375 level was associated with CTC positivity [169], suggesting that further investigations are needed to understand the complex network between YAP, ZEB1, miR375, EMT, and CTC formation. Additionally, hypoxia may, at least in part, induce EMT by stabilizing YAP and its nuclear translocation in PCa cell lines [170].

Not surprisingly, another study showed that inhibiting a key characteristic of epithelial tissue, namely, E-cadherin-mediated cell-cell interaction, resulted in EMT and increased dissemination of Madin–Darby canine kidney cells. Interestingly, dissemination could be partially prevented by YAP knockdown. The same study found that not only is YAP required to allow nuclear entry of the MET initiating Wilms tumor protein 1 (WT1), but both WT1 and YAP form a complex at the *CDH1* (E-cadherin) promoter and repress its transcription. These data, together with confirmation that E-cadherin inhibition upregulates YAP levels, indicate a double-negative feedback where E-cadherin and YAP mutually inhibit each other. This may be part of a switch between EMT and MET, thus potentially explaining the plasticity of the EMT process [171].

## 7. YAP Crosstalk with AR AKT and AR Pathways

Interestingly, one possible mechanism for PTEN loss of function is mediated by YAP. The pathway involves nuclear YAP-mediated activation of the TEAD family of transcription factors, leading to synthesis of the PTEN transcriptional repressor miRNA29c. Conversely, when YAP is inactivated via phosphorylation, PTEN levels are restored and the oncogenic function of YAP is inhibited [172]. Moreover, as mentioned above, PTEN ubiquitination can dephosphorylate and thus activate YAP causing its nuclear accumulation indicating a possible positive feedback regulation [158].

On the other hand, PTEN was identified as a negative regulator of AR activity such that the AR/PTEN interaction may mediate a tumor suppressor role for PTEN via suppression of AR and apoptosis induction in PCa cells [173]. However, as outlined above, the PTEN and AR network is still poorly understood, and data are conflicting. This is exemplified by another study with opposing findings, wherein PTEN deletion reduces both AR expression and AR transcriptional activity in PCa [131].

**Figure 4:**
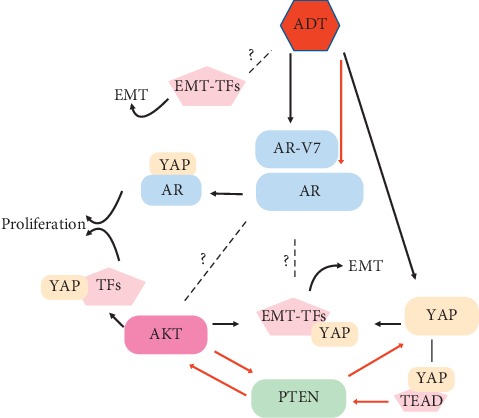
AR AKT and YAP interaction. Schematic presentation of reported and likely (dotted lines) network connections between ADT, AR, AKT, and YAP.

Taken together, emerging evidence indicates that YAP is part of the complex functional network that connects the AR and AKT pathways and thereby modulates PCa and mCRPC—at least in part—*via* EMT (Figure 4). However, more work is needed to better understand this interplay and its implications for the development of strategies to treat advanced PCa.

## 8. Analysis of PCa CTCs to Explore the AR-AKT-YAP Connection and EMT

The evaluation of molecular pathways underlying mCRPC is challenging because tissue biopsies are generally not available from late disease stages and animal models; further, although examination of tissue can provide some signaling pathway information, this mode of studying PCa has limitations. Liquid biopsies, and analysis of mCRPC CTCs, may be an alternative. While diagnostic CTC analysis in PCa is still in its infancy, there is ample evidence of its utility in this disease. Certainly, CTCs have been investigated by imaging and molecular technologies for expression of proteins, gene amplifications, mutations, and transcript expression on both targeted and comprehensive levels [174]. For PCa, increased CTC counts are associated with earlier disease progression and shorter OS, with enumeration of PCa CTCs using the CellSearch CTC platform gaining FDA approval as a prognostic indicator [175]. While common CTC isolation and analysis techniques favour epithelial CTCs, there have been numerous advances in improving capture, detection, and analysis of EMT-CTCs by screening for epithelial and mesenchymal marker expression [176–181]. Equally, as Table 1 shows, several major signaling pathways implicated in EMT have, to some extent, been analysed in CTC samples. In this review, we focussed on the AR, AKT, and Hippo pathways as being central to mCRPC, at least in part *via* EMT regulation. It is now important to consider how these pathways have been explored in CTCs, in order to gauge the potential for CTC analysis to advance our understanding of these pathways in mCRPC. Accordingly, we note that DNA-, RNA-, and protein-centric analyses for AR and AR-V7 levels in isolated CTCs have become a busy field of PCa research. Moreover, efforts are being made to translate CTC-based AR and AR-V7 detection into clinical settings aimed initially at stratifying patients to define either eligibility criteria or outcome markers for clinical trials (https://clinicaltrials.gov) [182].

mCRPC-associated AR amplification and mutation analysis have been performed in CTCs using hybridization techniques such as fluorescent *in situ* hybridization (FISH) and other molecular approaches. In general, these studies were able to validate the association of CTC-based AR amplification or mutation with mCRPC, while the relevance of AR cellular localisation in CTCs was shown in mCRPC and in response to taxanes [46, 47, 183–186]. The presence of full-length AR and AR-V7 in CTCs has been studied extensively at the RNA level and CTC-based AR-V7 in particular was found to correlate with mCRPC and primary resistance to abiraterone and enzalutamide [45, 182, 184, 187, 188]. Interestingly, there have also been efforts at detecting both AR and AR-V7 as biomarkers in other liquid biopsy entities, including plasma-derived circulating tumor RNA (ctRNA), exosomes, or even in urine. We recently compared some of these strategies and found both full-length AR and V7 RNA detection is more sensitive and specific if performed on CTC samples, as compared to ctRNA or exosomes. We also demonstrated that AR-V7 is detectable from CTC-RNA up to 48 h post blood draw into common EDTA vacutubes [189, 190]. With improved AR-V7-specific antibody availability, CTC immunocytostaining more recently revealed that specific detection of AR-V7 in CTC nuclei is an even better predictor of OS and PFS in CRPC patients [191, 192]. In general, it appears nuclear AR is found in most CTCs positive for AR-V7 RNA, reflecting the predominant tendency for AR-V7 to be nuclear localized in mCRPC tissue [188, 193]. In CRPC patients, AR-V7-positive CTCs have been shown to correlate with enzalutamide and abiraterone resistance [187]. In any case, when investigating the interplay of AR/AR-V7 with other pathways, especially transcriptional coactivators, immunocytodetection in CTCs appears to be the most logical strategy.

Several studies have also analysed PTEN loss in CTCs, which, as outlined above, may allow oncogenic activation of the AKT pathway and is an important PCa biomarker. Loss of *PTEN* and gain of AR copy numbers were reported in PCa CTCs [194–197], while testing for activation of the AKT pathway has been performed for example by phosho-Akt or phospho-S6 kinase immunostaining in breast cancer and multiple myeloma CTCs [198].

Reports on hippo signaling and YAP1 analysis in CTCs, by contrast, are still scarce. One study assessed expression of TAZ using RNA in situ hybridization (RNAish) probing of NSCLC CTCs. TAZ expression was detected more frequently in EGFR wild-type cancers while its expression in CTCs was associated with lymph node status of the disease [60]. It is likely that YAP1 could be analysed in a similar fashion in CTCs or preferentially using immunocytostaining, as the latter would also reveal cellular localisation and thus activity as well as colocalisation with other proteins. However, to our knowledge, direct detection of YAP1 in CTCs has not yet been reported, although the relationship of YAP1 to EMT suggests that activated YAP1 should correlate with increased formation of CTCs. Some indirect evidence lends further strength to this idea, as a recent report showed that the Rho GTPase activating protein 29 (ARHGAP29) is a transcriptional target of YAP1 in gastric cancer. High ARHGAP29 levels were shown to regulate cytoskeletal actin and cell migration. Importantly, the authors also demonstrated using a mouse model that CTCs exhibited increased ARHGAP29 RNA levels compared with primary tumor site cells [61, 199]. Final proof of a YAP1-ARHGAP29 connection in CTCs remains pending, however. Another transcriptional target of YAP is miR375 which was associated with CTC positivity, yet a direct connection was again not shown in CTCs [169].

Taken together, the reviewed data suggest that AR-AKT-YAP1 network can be analysed in CTCs. Since tumor tissue is rarely available in the mCRPC setting, and blood samples can be easily taken, future endeavours in CTC analysis could open the way to better understand ADT resistance and thereby inform the development of improved diagnostic, prognostic, and therapeutic capabilities.

Analysis of CTCs has provided a foundation for liquid biopsy, especially in the absence of biopsy tissue. However, there are serious challenges with CTC isolation, detection, and downstream analysis. One is that CTC numbers are relatively small within large populations of blood cells and the volume of blood that can be taken depends on the patient's general condition. CTCs are quite heterogeneous in terms of physical properties (size, elasticity, and surface charge), biological characteristics, and expression of different tumor markers making enrichment or isolation of all CTCs difficult (reviewed in [200]). In general, the low CTC numbers make downstream analysis of CTCs another challenge. Protein detection is usually limited to immunocytostaining which relies on antibody-based detection and the number of microscope channels available with 3 usually dedicated to detection of a CTC marker (often cytokeratin), a nuclear marker such as DAPI, and exclusion of a blood cell marker usually CD45. Nevertheless, some studies have detected additional proteins such as EMT markers [21, 22, 176] or posttranslational modifications such as phosphorylation of pFAK, pPI3K, pSRC, pEGFR, and pAkt [53, 201–204].

## 9. Conclusion

Here, we reviewed connections between the AR pathway and the AKT and Hippo pathways, exploring a potential role for this signaling nexus in EMT and mCRPC. Though current literature supports the importance of this tripartite relationship, further study is now needed to better evaluate its importance in PCa, as well as its clinical potential in defining biomarkers or drug targets. Analysis of PCa CTCs may facilitate deeper investigations into AR/AKT/Hippo pathway interactions, and how these drive EMT as well as ADT resistance. Such analyses may ultimately mediate the emergence of new diagnostic/prognostic assays directed towards PCa, though at this time insufficient data are available to establish feasibility of this concept. Indeed, while some aspects of these pathways have already been investigated in CTCs, optimisation of more comprehensive CTC analysis methods is now needed to permit the dissection of these pathway interactions, as a precursor to this significant goal.

## Figures and Tables

**Table 1:** Signaling pathways implicated in EMT and relevance to PCa.

Pathway	Implication in cancer-related EMT	Roles in PCa	CTC analysis
AR	Opposing data: elevation of AR expression and AR signaling in prostate tumors promotes PCa metastasis by induction of EMT [39]; other data suggest AR reverses EMT and ADT can induce EMT [40, 41]	Cell proliferation and tumor progression [42, 43]	Different AR expression patterns, amplification, mutation, and variant expression in PCa CTC [44–47]
AKT	PI3K-AKT directly or in crosstalk with other signaling pathways can induce EMT [48, 49]. Drugs inhibiting EMT *via* the Akt/GSK-3*β*/Snail pathway decrease the invasiveness of PCa cells [50]	Implicated in PCa cell proliferation and resistance to apoptosis [51, 52]	Phosphorylated EGFR and PI3K/Akt signaling kinases detected in breast cancer patient CTCs [53], pERK/Akt pathway in CTCs in hepatocellular carcinoma patients [54], PTEN loss in circulating tumor cells in CRPC patients [55]. No report in PCa CTCs
Hippo	Deregulation of the Hippo pathway contributes to EMT in colorectal cancer [56], and FZD2 could promote clinically relevant EMT in hepatocellular carcinoma involving Hippo pathway [57]	Emerging roles in PCa development, progression, EMT, and mCRPC [58, 59]	TAZ expression detected in NSCLC CTCs [60], YAP association with metastasis in human gastric cancer [61]. No report in PCa CTCs
MAPK	MAPK mediates epithelial-mesenchymal transition in cooperation with TGF-*β*/Smad2 signaling and increased Snail and Twist expression [62–64]	Linked to proliferation, early relapse, and development of mCRPC [65, 66]	MAPK gene expression signature shown in pancreatic CTCs [67], detection of mutant RAS and RAF in CRC and in melanoma CTCs [68, 69]. No report in PCa CTCs
NF-*κ*B	Hypoxia or overexpression of HIF-1*α* induces the EMT *via* NF-*κ*B in pancreatic cancer cells [70] and inhibition of NF-*κ*B deregulates EMT [71]	Promotes PCa cell survival, tumor invasion, metastasis, and chemoresistance [72, 73]	NSCLC-CTC gene expression profile was associated with cellular movement, cell adhesion and differentiation, and cell-to-cell signaling linked to PI3K/AKT, ERK1/2, and NF-*κβ* pathways [74]. No report in PCa CTCs
JAK/STAT	IFN-*γ* can induce epithelial-to-mesenchymal transition (EMT) in PCa cells *via* the JAK-STAT signaling pathway [75], and STAT3 may directly mediate EMT progression and regulate ZEB1 expression in CRC [76]	PCa progression, cell proliferation, and inhibition of apoptosis [51, 52]	No direct analysis of these pathways in CTCs
Wnt/*β*-catenin	Dysregulation of Wnt/*β*-catenin signaling has been implicated in the development of cancer in different tissues such as lung, skin, liver, and prostate [52], via regulating Zeb1 in CRC [77]	Wnt/*β*‐catenin pathway promotes the metastatic spread of prostate cancer cells by inducing EMT [78]	Epithelial type CTCs and activation of Wnt/*β*-catenin signaling in lung cancer cells [79]. No report in PCa CTCs
Notch	Crosstalk between the Jagged1/Notch and JAK/STAT3 signaling pathways by promoting EMT through Jagged-1 in ovarian cancer [80]	Notch signaling results in prostate tumor recurrence *via* EMT [81]	Increased production of ROS results in the upregulation of Notch1 in CTCs in metastatic breast and melanoma cancer [82]. No report in PCa CTCs

**Table 2:** EMT markers detected in PCa tissue.

Epithelial markers	Mesenchymal markers
E-cadherin [84]	Snail, Cat L [83]
	Vimentin, N-cadherin [84]
Cytokeratin [85]	Vimentin [85]
E-cadherin [88]	Twist [86, 87]
E-cadherin, cytokeratin [89]	N-cadherin [88]
